# Epithelial and Stromal MicroRNA Signatures of Columnar Cell Hyperplasia Linking Let-7c to Precancerous and Cancerous Breast Cancer Cell Proliferation

**DOI:** 10.1371/journal.pone.0105099

**Published:** 2014-08-14

**Authors:** Sofie Björner, Paul A. Fitzpatrick, Yaoyong Li, Craig Allred, Anthony Howell, Anita Ringberg, Håkan Olsson, Crispin J. Miller, Håkan Axelson, Göran Landberg

**Affiliations:** 1 Center for Molecular Pathology, Skåne University Hospital, Department of Laboratory Medicine Malmö, Lund University, Malmö, Sweden; 2 Breakthrough Breast Cancer Research Unit, Institute of Cancer Sciences, University of Manchester, Manchester Academic Health Science Centre, Paterson Institute for Cancer Research, The Christie National Health Service Foundation Trust, Manchester, United Kingdom; 3 Sahlgrenska Cancer Center, Department of Biomedicine, University of Gothenburg, Gothenburg, Sweden; 4 Cancer Research UK Applied Computational Biology and Bioinformatics Group, Paterson Institute for Cancer Research, Manchester, United Kingdom; 5 Department of Pathology and Immunology, Washington University School of Medicine, St Louis, Missouri, United States of America; 6 Department of Plastic and Reconstructive Surgery, SUS Malmö, Institute of Clinical Sciences Malmö, Lund University, Malmö, Sweden; 7 Department of Oncology, Skåne University Hospital, Institute of Clinical Sciences Lund, Lund University, Lund, Sweden; 8 Translational Cancer Research, Medicon Village, Department of Laboratory Medicine Malmö, Lund University, Lund, Sweden; King Faisal Specialist Hospital & Research center, Saudi Arabia

## Abstract

Columnar cell hyperplasia (CCH) is the earliest histologically identifiable breast lesion linked to cancer progression and is characterized by increased proliferation, decreased apoptosis and elevated oestrogen receptor α (ERα) expression. The mechanisms underlying the initiation of these lesions have not been clarified but might involve early and fundamental changes in cancer progression. MiRNAs are key regulators of several biological processes, acting by influencing the post-transcriptional regulation of numerous targets, thus making miRNAs potential candidates in cancer initiation. Here we have defined novel epithelial as well as stromal miRNA signatures from columnar cell hyperplasia lesions compared to normal terminal duct lobular units by using microdissection and miRNA microarrays. Let-7c were among the identified downregulated epithelial miRNAs and its functions were delineated in unique CCH derived cells and breast cancer cell line MCF-7 suggesting anti-proliferative traits potentially due to effects on Myb and ERα. MiR-132 was upregulated in the stroma surrounding CCH compared to stoma surrounding normal terminal duct lobular units (TDLUs), and overexpression of miR-132 in immortalized fibroblasts and in fibroblasts co-cultured with epithelial CCH cells caused substantial expression changes of genes involved in metabolism, DNA damage and cell motility. The miRNA signatures identified in CCH indicate early changes in the epithelial and stromal compartment of CCH and could represent early key alterations in breast cancer progression that potentially could be targeted in novel prevention or treatment schedules.

## Introduction

The initial model of the evolution of breast cancer was proposed to be a long process involving a few key stages, starting with proliferation and enlargement of the normal terminal duct lobular units (TDLUs) [1]. These alterations, denoted columnar cell hyperplasia (CCH), are common abnormalities in the adult female breast and are characterized by enlarged TDLUs lined by tightly packed columnar-shaped epithelial cells [2], [3]. The structures, referred to as atypical lobule of type A by Wellings, were suggested to represent the first precursor to ductal carcinoma *in situ* [1]. This assumption was later supported by associations between CCH and more advanced stages of breast carcinoma [2], [4], [5]. Characteristic features are increased number of oestrogen receptor α (ERα) positive cells, increased proliferation and decreased apoptosis [3], but the underlying mechanism of these changes has not been fully delineated.

The interplay between epithelial cells and the stromal compartment plays a significant role in breast development and cancer progression. Moreover, abnormal interactions between these compartments already at stages of early premalignant lesions of the breast have been suggested [6], [7]. The microenvironment consists of extra-cellular matrix (ECM) and different cell types including fibroblasts. These principal cellular components in the stroma are crucial in maintaining ECM homeostasis by synthesising and regulating degradation of the fibrillar components of the ECM. It is possible to observe changes in the stroma in early stages of cancer development, including an increased number of fibroblasts which have acquired an active phenotype that is also observed in wound healing [8]. These cells are commonly called cancer-associated fibroblasts and can promote tumour growth and progression [9].

MicroRNAs (miRNAs) are ∼21 nt long endogenous non-coding RNAs that regulate gene expression in a post-transcriptional manner mainly by interacting with the 3′UTR of their target mRNAs [10]. They play important roles during development, are involved in most cellular processes and have been implicated in cancer initiation and progression [11].

In this study we have identified miRNA expression signatures in both the epithelial and stromal compartment of CCH compared to TDLU that could represent key alterations in early breast cancer progression.

## Results

### CCH have altered miRNA expression patterns

**Figure 1:**
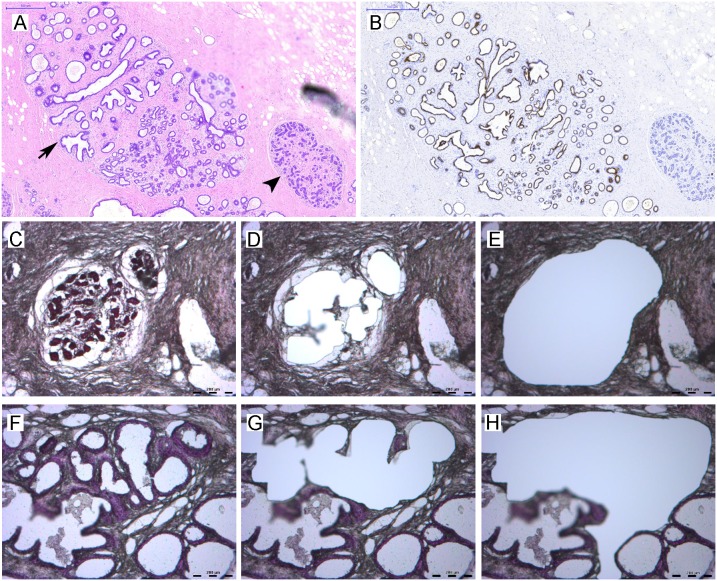
Characteristics of TDLU and CCH, and laser capture microdissection. Tissues were fixed in formalin, embedded in paraffin, sectioned and immunohistochemically stained with hematoxylin and eosin. Selected specimens were stained for ERα as additional control for the selection of the lesions. *A)* Identification of TDLU (arrow head) and CCH (arrow) based on morphology (microscopic evaluation) (20X). *B)* ERα expression in TDLU and CCH (20X). *C–H)* Images of TDLU (upper panel *C–E*) and CCH (lower panel *F–H*) before (*C, F)* and after laser capture microdissection (LCM) of epithelia (*D, G*) and stroma (*E, H*) (40X).

TDLUs and CCH with no or mild atypia from the same specimen were collected based on morphological evaluation and positive ERα expression using microdissection. Sufficient material for array analysis from epithelial cells (n = 4) and surrounding stroma (n = 2) were collected and RNA was isolated (Figure 1). MiRNA array analysis yielded expression data from 663 miRNAs. By comparing the expression in TDLUs and CCH we discovered 23 altered miRNAs in the epithelial compartment (n = 4, p<0.05, Table 1) and 17 in the surrounding stroma (n = 2, more than 2.0 fold change, Table 2). After moderating for false discovery rate, the number of significantly altered epithelial miRNAs decreased to two. Most miRNAs, 22 in the epithelial compartment and 14 stroma, were downregulated. The selected epithelial and stromal miRNA in this study was downregulated and upregulated, respectively, in CCH compared to TDLU.

**Table 1:** MiRNA expression signatures in epithelial and stromal compartments of CCH compared to TDLU.

Epithelial cells (n = 4)	Fold change (log 2)	*P* †	q-value (FDR)
hsa-miR-625	−0.79	<0.001	0.046
hsa-miR-455-5p	−1.75	0.001	0.046
hsa-miR-532-3p	−0.78	0.004	0.061
hsa-miR-92a	−1.46	0.004	0.061
hsa-miR-886-3p	−1.43	0.006	0.061
hsa-let-7c	−1.17	0.006	0.061
hsa-miR-383	−2.36	0.007	0.061
hsa-miR-505*	−1.02	0.010	0.083
hsa-miR-150	−1.34	0.017	0.086
hsa-miR-29b	−1.04	0.017	0.086
hsa-miR-27a	−0.93	0.017	0.086
hsa-miR-491-5p	−1.35	0.017	0.086
hsa-miR-190b	+1.61	0.014	0.086
hsa-miR-130a	−1.18	0.021	0.094
hsa-miR-886-5p	−1.26	0.025	0.094
hsa-miR-204	−1.30	0.026	0.094
hsa-let-7f	−1.54	0.028	0.094
hsa-miR-335*	−1.23	0.022	0.094
hsa-miR-20a*	−1.20	0.027	0.094
hsa-miR-135a*	−0.63	0.029	0.094
hsa-miR-494	−1.24	0.040	0.125
hsa-miR-26b*	−1.14	0.043	0.128
hsa-miR-29c	−0.87	0.046	0.129

Results are presented as log2 fold change.

† *P* was calculated using paired two-sided Student’s t-test.

The false discovery rate (FDR) was calculated from the p-values of the 137 miRNAs whose fold changes between the two conditions were greater than 1.5 and is presented as q-values.

**Table 2:** MiRNA expression signatures in the stromal compartment of CCH compared to TDLU.

Stroma (n = 2)	Fold change (log 2)‡
hsa-miR-539	+4.82
hsa-miR-132	+2.61
hsa-miR-221	+2.34
hsa-miR-135b	−2.15
hsa-miR-451	−2.19
hsa-miR-642	−2.23
hsa-miR-130b	−2.32
hsa-miR-20a	−2.35
hsa-miR-204	−2.35
hsa-miR-192	−2.40
hsa-miR-29b	−2.43
hsa-miR-452	−2.48
hsa-miR-124	−2.84
hsa-miR-95	−2.94
hsa-miR-423-5p	−2.97
hsa-miR-148a	−2.98
hsa-miR-139-3p	−3.40

Results are presented as log2 fold change.

‡ A fold change ≥2.0 was used as cut-off.

### Involvement of let-7c in epithelial CCH features

**Figure 2:**
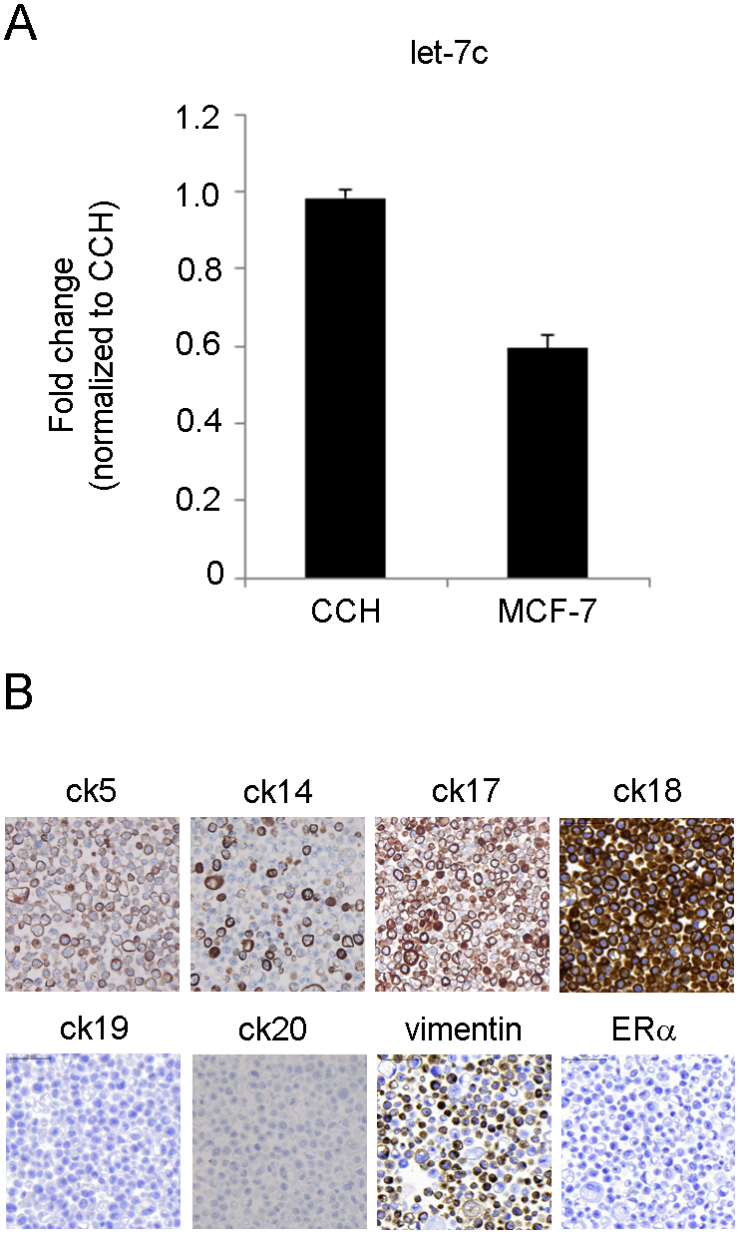
Characteristics of CCH and MCF-7 cells. *A)* Baseline endogenous levels of let-7c were lower in CCH cells compared to MCF-7 cells, modelling the observed levels in TDLU and CCH in patients (n = 2, p = 0.072). *B)* CCH cells were ER-negative and combined luminal and basal epithelial phenotypes (80X).

**Figure 3:**
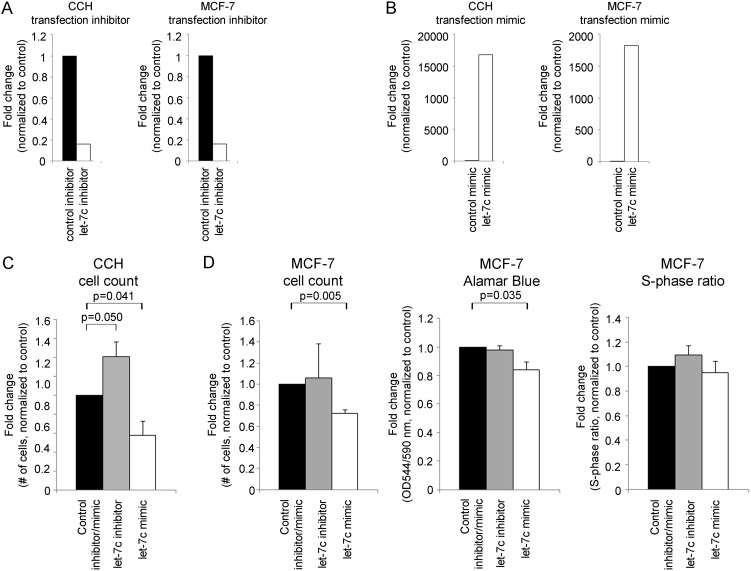
The effect of let-7c on proliferation in CCH cells and MCF-7 cells. Modulation of miRNA levels by *A)* inhibitors and *B)* mimics was validated and confirmed by qRT-PCR. *C)* Proliferation measured by cell count performed in CCH cells after miRNA level alteration. *D)* Proliferation measured by cell count, Alamar Blue and cell cycle analysis (S-phase ratio) in MCF-7 cells after miRNA level alteration. All results are calculated using two-sided paired t-tests and presented as mean plus standard deviation, n = 3.

Epithelial cells in CCH lesions have elevated ERα expression, decreased apoptotic rate and increased proliferation compared to cells in TDLUs [3]. We chose to study the involvement of one selected miRNA, let-7c, in the regulation of these characteristics in CCH. Let-7c was chosen from the array data based on previous reports linking it both to proliferation and apoptosis [12], [13]. Let-7c was also predicted to target the transcription factor Myb that was previously shown to be upregulated in CCH lesions [14]. We used cells derived from CCH lesions and the ER positive breast cancer cell line MCF-7. The CCH cells were chosen based on their origin and MCF-7 was selected as a model for ER positive epithelial cells at a more progressed stage and expressed lower levels of let-7c compared to the CCH cells. (Figure 2 and figure S1) The decreased expression of let-7c was confirmed in one additional patients using microdissection and qRT-PCR (figure S2). Using miRNA inhibitors and mimics, we modulated the expression levels of let-7c in the CCH and MCF-7 cells (Figure 3A–B). We observed that decreased levels of let-7c in CCH cells significantly increased the number of cells whereas the opposite effect was observed after let-7c overexpression (p = 0.050 and p = 0.041, respectively, Figure 3C). This was confirmed in MCF-7 cells where an increased expression of let-7c significantly decreased the number of cells and the proliferation rate measured by Alamar Blue (p = 0.005 and p = 0.035, respectively, Figure 3D). However, we did not detect any significant differences in S-phase ratio after modulation of let-7c levels.

**Figure 4:**
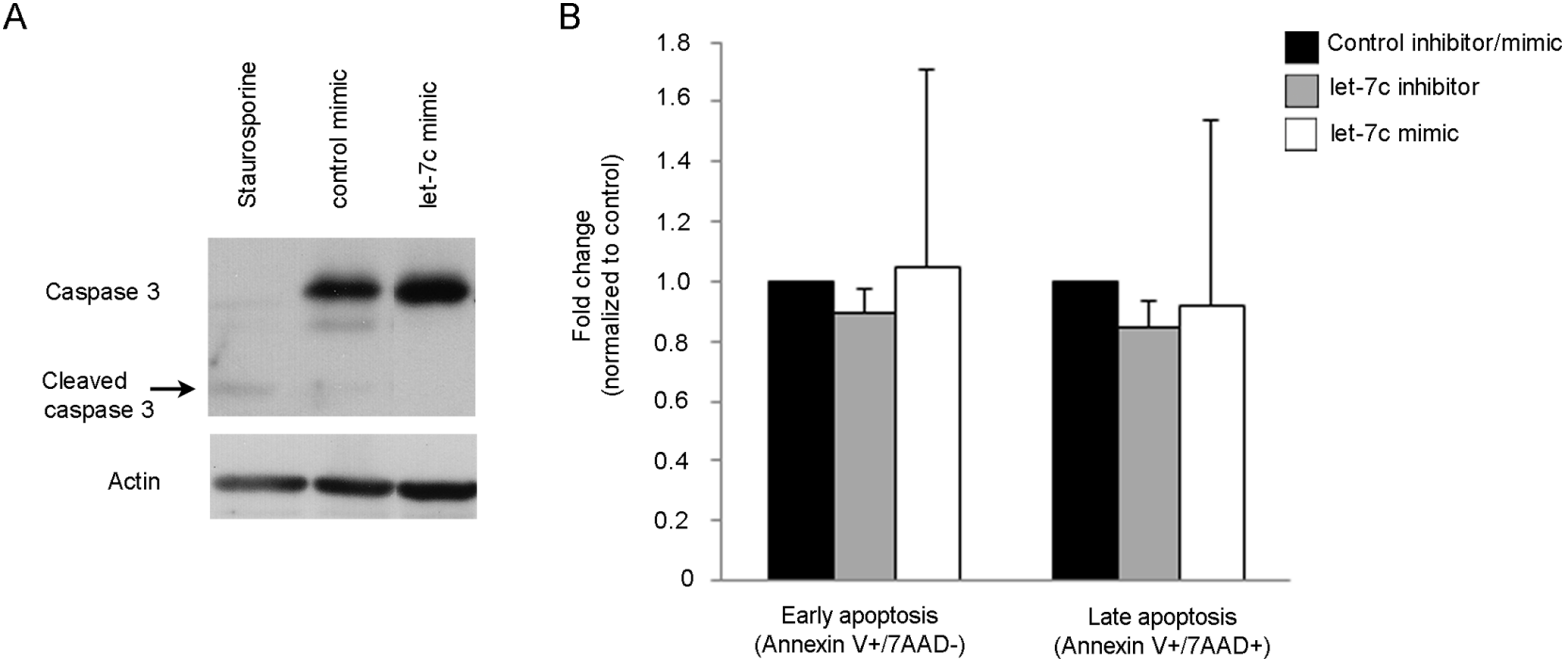
The effect of let-7c on apoptosis in CCH cells and MCF-7 cells. *A)* Apoptosis analysis of CCH cells after alteration of miRNA levels measured by cleaved caspase 3 protein expression and Western blot analysis. CCH cells treated with 1 µM staurosporine were used as a positive control for apoptosis. *B)* Early (Annexin V+/7AAD–) and late (Annexin V+/7AAD+) apoptosis was measured using flow cytometry in MCF-7 cells (n = 3).

In order to see if the observed effects on cell number were indeed a measurement of proliferation, we investigated the possible involvement of apoptosis. We did not observe the apoptotic marker cleaved caspase 3 in the CCH cells after let-7c overexpression (Figure 4A), nor did we observe any differences in either early apoptosis (Annexin V+/7AAD–cells) or late apoptosis (Annexin V+/7AAD+cells) in MCF-7 cells (Figure 4B).

### Links between let-7c, Myb and ER

**Figure 5:**
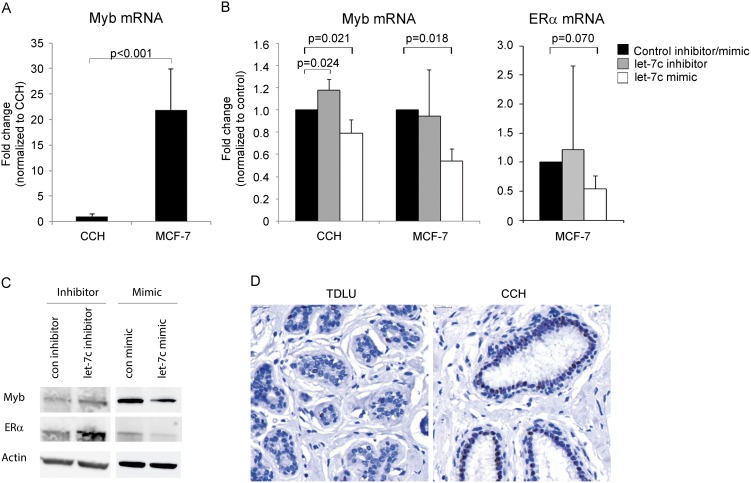
Associations between let-7c and Myb and ERα. *A)* Endogenous Myb mRNA levels, measured by qRT-PCR in CCH and MCF-7 cells. Results are calculated using two-sided unpaired t-test and presented as mean plus standard deviation, n>3. *B)* Myb mRNA levels were measured with qRT-PCR after up- and downregulation of let-7c in CCH cells and MCF-7 cells. ERα mRNA was measured in MCF-7 cells after let-7c modulation. Results are calculated using two-sided paired t-tests and presented as mean plus standard deviation, n = 3. *C)* Myb and ERα protein expression measured by Western blot after let-7c downregulation and upregulation. The figure shows representative blots from one of three independent experiments. *D)* Protein expression of Myb in TDLU and CCH (80X).

In order to investigate possible gene targets for let-7c, we used gene expression data from a previous study comparing epithelial gene expression differences between epithelial cells in TDLUs and CCH [14]. The data was related to potential target sites for let-7c using a target prediction algorithm [15], [16]. Interestingly, the transcription factor Myb, which had elevated levels in CCH in the reported study, displayed a potential target site for let-7c (data not shown). In line with that, we detected lower levels of Myb in the CCH cells compared to MCF-7 cells (p<0.001, Figure 5A). In order to investigate the relation between let-7c and Myb expression, we modulated the levels of let-7c and monitored the levels of Myb mRNA. Decreased expression of let-7c resulted in significantly increased levels of Myb mRNA in the CCH cells (p = 0.024), and the opposite was observed after upregulation of let-7c in both CCH cells and MCF-7 cells (p = 0.021 and 0.018, respectively). A similar pattern was also observed for ERα (p = 0.070) after overexpression of let-7c, and the link between Myb and ERα is well-established (Figure 5B) [17], [18]. We also detected elevated protein levels of Myb and ERα in MCF-7 cells after downregulation of let-7c, and the opposite effect after upregulation of let-7c (Figure 5C). In addition, we detected elevated Myb protein expression in CCH lesions compared to normal TDLUs in human mammary tissue, validating the experimental *in vitro* findings *in vivo* and are in agreement with the published data [14] (Figure 5D).

### Interactions between epithelial and stromal compartments

The almost 3-fold increase of miR-132 in the stroma surrounding CCH lesions is interesting due to the essential role of stromal miR-132 in the development of ductal structures in mouse mammary glands [19]. We therefore increased the levels of miR-132 in human mammary fibroblasts and performed gene expression array analyses. The top ten up- and downregulated gene candidates are presented in table 3 and include *FBN3*, *COL9A1* and *UBE3A*. Several of the identified gene candidates were involved in pathways regulating metabolism, DNA damage response, cell motility and the cytoskeleton.

**Table 3:** Gene expression analysis of miR-132 overexpressing fibroblasts.

10 most upregulated genes	Fold change(log2)	10 mostdownregulatedgenes	Fold change (log2)
IQCH	3.91	GART	−1.87
UBE3A	3.32	LUZP2	−1.85
FBN3	2.92	PXK	−1.83
INTS2	2.82	USP34	−1.82
NLRP3	2.72	APC2	−1.80
RRM2	2.69	NFE2L2	−1.79
COL9A1	2.67	MYO5C	−1.77
ARID4A	2.65	KIAA1324	−1.76
SFMBT1	2.60	GBAS	−1.75
AIM1	2.49	TFIP11	−1.72
**Top 10 upregulated pathways**	*P* †	Ratio ‡	Genes
Hereditary Breast Cancer Signaling	0.003	3/129	FANCM, PIK3C2G, FANCC
Fatty Acid Metabolism	0.003	3/184	ACADSB, ACADM, CYP3A5
β-alanine Metabolism	0.006	2/93	ACADSB, ACADM
Role of BRCA1 in DNA Damage Response	0.009	2/61	FANCM, FANCC
Propanoate Metabolism	0.009	2/121	ACADSB, ACADM
Valine. Leucine and Isoleucine Degradation	0.011	2/106	ACADSB, ACADM
Role of Pattern Recognition Receptors inRecognition of Bacteria and Viruses	0.016	2/87	NLRP3, PIK3C2G
TR/RXR Activation	0.019	2/96	NCOA2, PIK3C2G
Estrogen Receptor Signaling	0.040	2/136	NCOA2, ESR2
AMPK Signaling	0.043	2/167	PIK3C2G, PFKFB2
**Top 10 downregulated pathways**	*P* †	Ratio ‡	Genes
Molecular Mechanisms of Cancer	0.003	8/377	SMAD2, PAK3, PRKAR2A, MDM2, RALBP1, RASA1, RALGDS, FNBP1
Regulation of Actin-based Motility by Rho	0.003	4/91	WASL, PAK3, BAIAP2, FNBP1
Actin Cytoskeleton Signaling	0.005	6/238	MYH6, WASL, PAK3, APC2, DIAPH3, BAIAP2
Pancreatic Adenocarcinoma Signaling	0.007	4/119	SMAD2, MDM2, RALBP1, RALGDS
Mitotic Roles of Polo-Like Kinase	0.010	3/64	SLK, PPP2CB, CDC16
Angiopoietin Signaling	0.012	3/74	PAK3, STAT5B, RASA1
Purine Metabolism	0.013	6/390	NME4, MYH6, KIF1B, PDE8B, RALBP1, GART
Integrin Signaling	0.015	5/209	WASL, PAK3, ASAP1, CTTN, FNBP1
Complement System	0.024	2/35	CFB, C2

Results are presented as log2 fold change.

† *P* was calculated by the Ingenuity System.

‡ Ratio: altered genes/total #genes in pathway.

To investigate whether elevated levels of miR-132 in the stroma also affected human mammary epithelial cells, we co-cultured epithelial CCH cells with fibroblasts overexpressing miR-132 and performed gene expression array analysis of the epithelial cells (Table 4). Interestingly, we observed approximately 450 significantly altered genes including metabolic genes (*GLUL*, *ACSS2*, *DHRS9*), genes involved in protein turn-over (*MARCH9*, *CUL4B*), genes implicated in the cytoskeleton and cell motility (*IFFO1*, *DOCK5*), genes encoding cell cycle regulatory proteins (*NEK11*, *NEK9*), as well as genes related to the main features of CCH: proliferation (*RASGRP3*), apoptosis (*WNK3*) and ERα expression (*GLUL*). The pathway analysis further revealed that pathways involved in cytoskeleton and tight junction signalling via isoforms of the myosin heavy chain (*MYH*) gene were upregulated.

**Table 4:** Gene expression analysis of epithelial CCH cells after co-culture with miR-132 overexpressing fibroblasts.

15 most upregulated genes	Fold change(log2)	15 mostdownregulatedgenes	Fold change (log2)
TFEC	5.90	NEK9	−5.53
MARCH9	3.95	SLMAP	−4.98
EPB41L3	3.92	DOCK5	−4.96
DNAH11	3.60	CUL4B	−4.80
NEK11	3.45	AP3B1	−4.79
IFFO1	3.41	C1orf9	−4.78
GLUL	3.40	PLEKHA5	−4.72
TAF1C	3.40	ZZEF1	−4.65
ACSS2	3.32	DHRS9	−4.64
NCRNA00114	3.30	BZW2	−4.61
WNK3	3,30	ULK2	−4,49
TRMT2B	3,22	SLTM	−4,44
PIWIL3	3,16	ADAMTS16	−4,40
MAP2	3,16	POT1	−4,31
RASGRP3	3,13	TBCK	−4,30
**Significantly upregulated pathways**	*P* †	Ratio‡	Genes
Cellular Effects of Sildenafil (Viagra)	0.001	5/151	SLC4A5, MYH2, MYH8, ITPR1, MYH1
Calcium Signaling	0.012	4/207	MYH2, MYH8, ITPR1, MYH1
Actin Cytoskeleton Signaling	0.023	4/238	MYH2, MYH8, NCKAP1L, MYH1
Hepatic Fibrosis/Hepatic Stellate CellActivation	0.035	3/147	MYH2, MYH8, MYH1
Assembly of RNA Polymerase I Complex	0.045	1/13	TAF1C
Tight Junction Signaling	0.046	3/164	MYH2, MYH8, MYH1
**Significantly downregulated** **pathways**	*P* †	Ratio‡	Genes
Glycerophospholipid Metabolism	0.003	7/179	GPAM, PLCB2, BCHE, DGKB, DGKG, LPIN2, LYPLA1
Glycerolipid Metabolism	0.005	6/148	GPAM, DHRS9, LIPF, DGKB, DGKG, LPIN2
Phospholipid Degradation	0.008	5/93	PLCB2, DGKB, DGKG, LPIN2, LYPLA1
Cellular Effects of Sildenafil (Viagra)	0.022	6/151	MYH4, PLCB2, ADCY2, CACNA1E, PDE4C, MYH7
G-Protein Coupled Receptor Signaling	0.024	15/528	PLCB2, ADCY2, CCKAR, PDE4C, OPN1LW, SOS2, PDE6C, GPR107, HTR6, RGS12, GPR64, CAMK2D, DRD1, RXFP1, BAI1
cAMP-mediated signaling	0.025	8/218	ADCY2, CAMK2D, PDE4C, DRD1, PDE6C, CNGB1, HTR6, RGS12
Purine Metabolism	0.033	9/392	ADCY2, POLR3B, PDE4C, KIF1B, NUDT9, PDE6C, MYH7, REV3L, RALBP1

† *P* was calculated by the Ingenuity System.

‡ Ratio: altered genes/total #genes in pathway.

Results are presented as log2 fold change.

### Case study – progression of TDLU via CCH to invasive breast cancer

**Figure 6:**
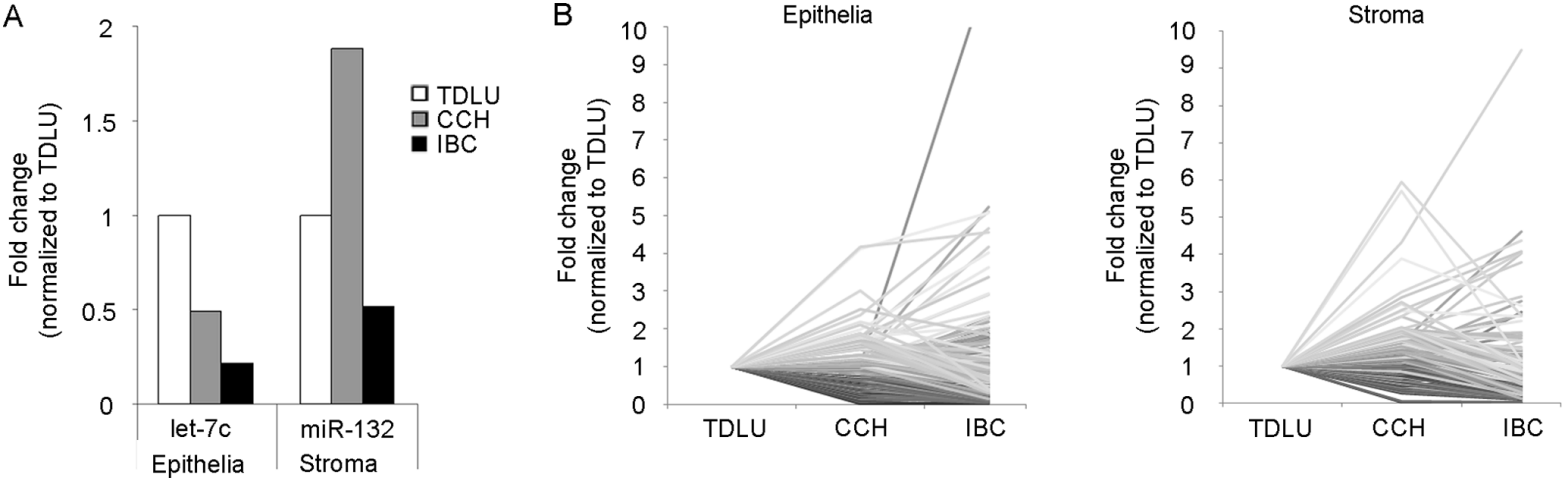
MiRNA expressions in TDLU, CCH and invasive breast cancer (IBC) in one case study. *A)* Expression levels of the selected epithelial and stromal miRNA in TDLU, CCH and IBC. *B)* Line charts illustrating the distribution of fold change expressions for 663 miRNAs in the epithelial and stromal compartment respectively.

To further illustrate the fluctuation of miRNA levels in breast cancer progression, we analysed the miRNA expression profile of the epithelial and stromal compartments of another patient who displayed invasive breast cancer (IBC) in addition to TDLUs and CCH. Initially, we examined whether the selected miRNAs followed the observed pattern noticed in CCH lesions (Figure 6A). The expressions of the epithelial miRNA let-7c continued to decrease in IBC, whereas the expression level of the stromal miR-132 in the IBC was even lower than in the stroma surrounding normal TDLUs suggesting that in this case, miR-132 was only overexpressed in the early precancerous lesion.

We also investigated if there was a tendency for a certain expression pattern to be predominant in the progression towards IBC and plotted the fold change expressions for all miRNA as illustrated in figure 6B. The two most common patterns in both the epithelial and stromal compartment were progressive decrease in miRNA expression (TDLU>CCH>IBC) including 19% of all miRNAs in the epithelia and 24% in the stroma, and downregulation of the miRNA in CCH with a similar level in IBC (TDLU>CCH = IBC) comprising 25% in the epithelia and 16% in the stroma. We also observed a more than 10-fold increased expression of miR-652 in the epithelial compartment and of miR-484 in the stromal compartment of the sample with invasive breast cancer.

## Discussion

**Figure 7:**
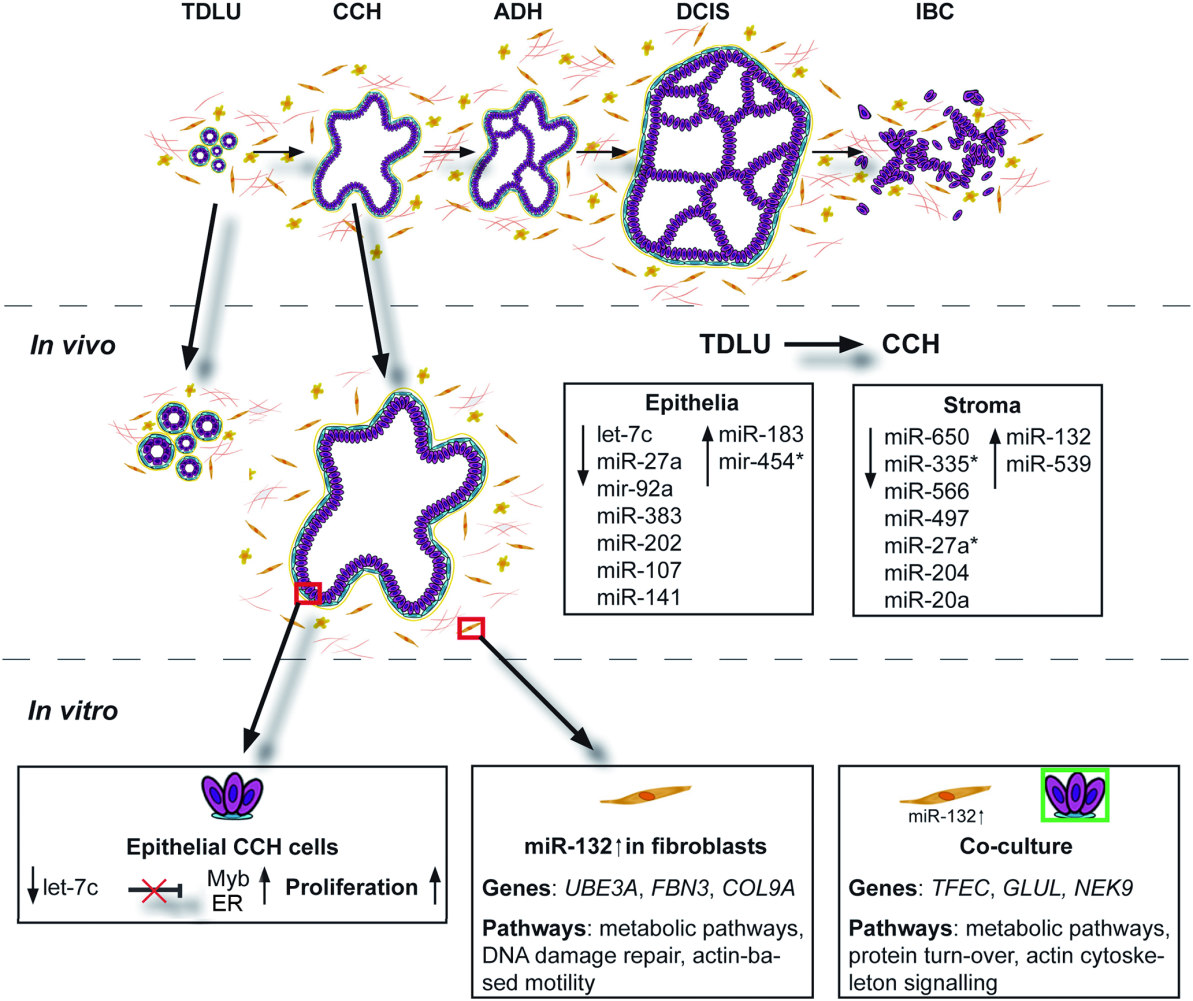
Summary of the reported study. The study included *in vivo* obtained epithelial and stromal miRNA expression signatures and subsequent *in vitro* studies linking let-7c to proliferation, Myb and ERα. MiR-132 upregulation in fibroblasts affected several pathways as well as greatly influenced co-cultured epithelial CCH cells.

By using microdissection we have identified differences in miRNA expression in both epithelial and stromal compartments of CCH, the proposed lesion to be the first histologically recognizable alteration in the progression towards breast cancer, compared to TDLU. The main findings are summarized in figure 7. The majority of miRNAs were downregulated, which could possibly explain the overall upregulation of genes observed by Lee *et al* in epithelial CCH cells *in vivo* [14]. Among the identified downregulated miRNAs in the epithelial compartment was let-7c which appeared to play a role in one of the main characteristics of CCH; increased proliferation. Let-7c has been reported to be downregulated in pancreatic cancer, and in prostate cancer it has the ability to inhibit growth both *in vitro* and *in vivo* [12], [20]. We observed that let-7c had anti-proliferative properties but was not involved in apoptosis as previously described [13]. One of the *in silico* predicted targets for let-7c was the proliferation promoting transcription factor Myb which has been shown to be overexpressed in colon and breast cancer and our experimental data support the notion that let-7c has a negative effect on Myb mRNA and protein expression in both CCH cells and MCF-7 cells [21]. We also observed similar effects on ERα, which could explain the noticed effects on Myb in MCF-7 [17], [18]. However, the *in silico* miRNA target prediction algorithm did not predict let-7c to target ERα, and the CCH cells are ER negative, suggesting that let-7c can probably indirectly affect both Myb and ERα independently. Based on these results, one could conclude that let-7c has negative effects on cell proliferation and a most likely indirectly negative effect on Myb and ERα expression, however the exact link remains to be investigated.

In the stromal compartment, we observed an increase of miR-132. This is interesting since stromal miR-132 is crucial in the development of the mouse mammary gland [19]. By overexpressing miR-132 in fibroblasts, we indeed observed several changes. Among the identified upregulated candidate genes was *UBE3A*. UBE3A is an E3 ubiquitin ligase targeting the CDK inhibitor p27 in mouse brain, and downregulation of UBE3A led to cell cycle arrest [22]. This effect on proliferation can possibly link *UBE3A* to the increased proliferative rate of cancer-associated fibroblasts observed in prostate cancer [23]. Additionally, one of the altered pathways was “Pancreatic Adenocarcinoma Signalling” which is in line with previous studies associating pancreas cancer and miR-132 [24], [25]. To see if the increased expression of miR-132 in the stroma had any effect on the epithelial CCH cells, we tried to mimic this scenario by overexpressing miR-132 in fibroblasts and then co-cultured them with epithelial CCH cells followed by gene expression of the epithelial cells. Among the altered genes were *GLUL*, *WNK3* and *RASGRP3*. They have all respectively been associated with at least one of the main characteristics of CCH. *GLUL* encodes for glutamine synthetase (GS) and is overexpressed in ER positive luminal breast cancer subtypes and cell lines compared to the basal subtype and cell lines [26]. We observed an increase in *GLUL* expression which could possibly indicate a progression of the epithelial CCH cells to a more ER positive luminal carcinogenic phenotype. Increased levels of ERα observed in CCH *in vivo* further indicate this possible progression. We also observed elevated expression of one of the members of the kinase subfamily WNK, *WNK3*. *WNK3* promotes cell survival in HeLa cells by delaying the apoptotic response in a caspase-3-dependent manner [27]. The observed upregulated gene *RASGRP3* is a Ras activator that has been reported to be elevated in human melanoma. Upregulation of RASGRP3 in melanocytes increased the cell proliferation and made the cells tumorigenic in a mouse xenograft model [28].

Moreover, we studied the miRNA expression profiles in one patient who displayed invasive breast carcinoma in addition to TDLU and CCH. The most prominent expression pattern was a continuous decrease of expression or a sustained low level even in the breast cancer sample. This is in line with earlier publications illustrating downregulation of miRNAs in the progression towards cancer and supports a general inhibitory function for miRNAs [29]. Interestingly, approximately 16% of all miRNAs in the epithelia had minor or no change between the different stages whereas in the stroma this pattern was only observed in 4% of the miRNAs, a possible indication of high stromal activity in early precancerous lesions [30], underlining the significance of studying these two compartments separately. We also observed that two miRNAs in the different compartments had a more than 10-fold increased expression in the invasive breast cancer sample; miR-652 in the epithelial compartment and miR-484 in the stroma. Decreased levels of miR-652 in blood was recently reported to be a biomarker for luminal A-like breast tumours [31], and increased expression of miR-484 has been observed in serum of early breast cancer patients [32].

Taken together, the identified epithelial and stromal miRNA changes may represent very early important changes in breast cancer progression that might be targeted in future prevention schedules.

## Materials and Methods

### Patient samples and cells

Specimens were obtained from women between 37–51 years of age at the time for prophylactic mastectomy at Malmö University Hospital (1995–2008). Five patients were included in the comparison between TDLU and CCH; two patients had *BRCA1* and one had *BRCA2* mutation without history of breast cancer, one had both personal and family history of breast cancer without identified mutation, and one had a therapeutic mastectomy due to lobular invasive carcinoma and simultaneous contralateral prophylactic mastectomy without family history of breast cancer. The patient in the cancer progression case study had a family history of breast cancer but no identified *BRCA1/2* mutation. The patients’ written and verbal consent to participate in this study was registered and the study was approved by the Ethics Committee at Lund University, Sweden.

Human mammary CCH cells were derived from a clinical sample after written informed consent and approved by the Institutional Review Board for Baylor College of Medicine and Affiliated Hospitals, Houston, Texas. Cells were cultured in DMEM-F12 supplemented with 5% horse serum, cholera toxin (100 ng/ml), hydrocortisone (0.5 µg/ml), insulin (10 µg/ml), EGF (20 ng/ml) and 1% penicillin-streptomycin. Human breast cancer cell line MCF-7 was purchased from ATCC and grown in DMEM growth medium supplemented with 10% fetal bovine serum (FBS), 1% glutamine, 1x non-essential amino acids and 1x streptomycin and penicillin. The normal human GFP-tagged immortalized mammary fibroblasts cell line 218TGpp was a kind gift of Professor Akira Orimo (Stromal-Tumour Interaction Group, Paterson Institute for Cancer Research, The University of Manchester) [33]. Cells were grown in DMEM supplemented with 10% fetal bovine serum and 1% penicillin-streptomycin. All cells were maintained at 37°C in a humified incubator (5% CO_2_).

### Laser capture microdissection and RNA extraction

Epithelial cells and surrounding stroma from paired specimens containing sufficient material of both normal TDLUs and CCH were collected using laser capture microdissection (Leica Microsystems AB, Wetzlar, Germany). Tissue from both epithelial and stromal compartments of TDLUs, CCH and invasive breast cancer was collected from the cancer progression case study patient. Total RNA was extracted using RecoverAll (Ambion/Life Technologies, Carlsbad, CA) following the manufacturer’s instructions.

### TaqMan MicroRNA Arrays

MiRNA expression profiles were obtained using TaqMan MicroRNA Arrays v2.0 A and B (Applied Biosystems/Life Technologies, Carlsbad, CA). 25 ng of total RNA was reverse-transcribed using Megaplex Primer Pools A and B followed by preamplification with Megaplex PreAmp Primers A and B. The arrays were run on the 7900 HT Fast Real-Time System according to manufacturer’s instructions (Applied Biosystems/Life Technologies). Data analysis was performed using DataAssist v1.0 (Applied Biosystems/Life Technologies) and determined using the comparative threshold cycle (Ct) method with RNU48 and MammU6 as endogenous controls.

### Quantitative real-time PCR

Individual TaqMan MicroRNA Assays (Applied Biosystems/Life Technologies) were used for quantification of let-7c, miR-132 and the endogenous controls RNU48 and MammU6 according to the manufacturer’s instructions. *SDHA*, *UBC*, and *YWHAZ* were used as reference genes for *Myb* and *ERα* quantification. Total RNA was extracted using the miRNeasy kit (Qiagen, Hilden, Germany) according to the manufacturer’s instructions with the exception of replacing chloroform with 70 µl BCP (1-bromo-3-chloro-propan). For miRNA quantification, 500 ng of RNA was reverse-transcribed using Megaplex Primer Pool A. For mRNA quantification, 2 µg of RNA was converted to cDNA using High Capacity cDNA Reverse Transcription Kit (Applied Biosystems/Life Technologies). qRT-PCR was carried out using 7300 Real Time PCR System (Applied Biosystems/Life Technologies). The Ct method was used for calculation of gene expression.

### Transfections

Cells were transfected with 20 nM miRNA Inhibitor (Exiqon) or 25 nM miRIDIAN miRNA mimics (Dharmacon/Thermo Fisher Scientific, Waltham, MA) or corresponding non-targeting control oligonucleotides of the same length, according to the manufacturer’s instructions using Lipofectamine 2000 (Invitrogen/Life Technologies, Carlsbad, CA) in Opti-MEM medium depleted from serum and penicillin-streptomycin. The medium was changed to serum-containing medium 5 h after transfection.

### Cell proliferation assays

Cell number was measured by counting cells 48 h after transfection using a Bürker chamber and by using the Moxi Automated Cell Counter (ORFLO Technologies).

Cell proliferation was monitored using Alamar Blue (Invitrogen). Cells were seeded out in 96 well plates at 10000 cells/well) 24 h prior to transfection. Cell viability was measured after 48 h by adding 2% Alamar blue and fluorescence was read at 544/590 nm after 1 h incubation.

### Flow cytometry for cell cycle and apoptosis analysis

Cell cycle analysis was performed using propidium iodide as previously described [34].

For apoptosis analysis, cells were stained with Annexin V-FITC and 7AAD (BD Pharmingen, San Jose, CA) to analyse both early (Annexin V+/7AAD–) cells and late (Annexin V+/7AAD+) apoptotic cells, and analyzed with BD Accuri C6 Flow Cytometer (BD Biosciences, San Jose, CA). Data analyses were performed with FlowJo (FlowJo, Ashland, OR).

### Immunohistochemistry and western blot

Immunohistochemistry was performed using Dako’s Autostainerplus with the EnVisionFlex High pH-kit (DAKO, Glostrup, Denmark) with the following antibodies: CK5 (Novocastra, Wetzlar, Germany), CK14 (Novocastra), CK17 (DAKO), CK18 (DAKO), CK19 (DAKO), CK20 (DAKO), vimentin (DAKO), ERα (DAKO), Myb (Epitomics, San Francisco, CA). Western blot was performed as previously described [35] using the following primary antibodies: caspase 3 (Cell Signaling Technology, Danvers, MA), β-actin (Santa Cruz Biotechnology, Santa Cruz, CA), Myb (Merck Millipore clone 1-1, Darmstadt, Germany), ERα (DAKO).

### Co-cultivation of cells and *fluorescence-activated cell sorting* (FACS)

GFP-tagged fibroblasts were transfected with either control or miR-132 mimics. Cells were harvested 24 h after transfection and equal number of fibroblasts and low passage CCH cells were seeded together in CCH cell medium and incubated for 48 h. Cells were labelled with 7AAD in order to sort out live cells before the GFP positive fibroblasts and GFP negative CCH cells were separately collected using FACS followed by RNA extraction from the CCH cells.

### CGH analysis, statistical methods and gene expression profiling

CGH analysis was performed on the Agilent G3 Human 1×1 m CGH Microarray by ATLAS Biolabs GmbH in Berlin, Germany. The list of potentially significantly altered miRNAs in the epithelial cells and statistical significance to all *in* ****** *vitro* experiments were calculated using two-sided Student’s T-tests. The R package qvalue was used to compute the false discovery rate [36]. The Affymetrix GeneChip Human Gene 1.0 ST Arrays were performed by AROS Applied Biotechnology A/S (Aarhus, Denmark) and analysed with R, Bioconductor and Ingenuity Systems (Redwood City, CA). The RMA was used to normalise and summarise the expression values at the probeset level [37]. Probesets were mapped to genes using the Bioconductor package annmap to position probesets relative to Ensembl version 60 annotations [38]. The R package Limma was used to find differentially expressed genes [39] and Ingenuity to find the enriched pathways from the differentially expressed gene lists. The raw array data is located at the GEO database with the accession number: GSE46199.

## Supporting Information

Figure S1 **CGH analysis of CCH cells.**
(TIF)

Figure S2 **Validation of miRNA microarray results.** The expression of let-7c was analysed in microdissected epithelial tissue from one additional patient using qRT-PCR.
(TIF)
