# Supplementary figures and images for: Epithelial and Stromal MicroRNA Signatures of Columnar Cell Hyperplasia Linking Let-7c to Precancerous and Cancerous Breast Cancer Cell Proliferation
Source: PLoS One. 2014 Aug 14;9(8):e105099. doi: 10.1371/journal.pone.0105099 (PMC4133372; doi:10.1371/journal.pone.0105099)

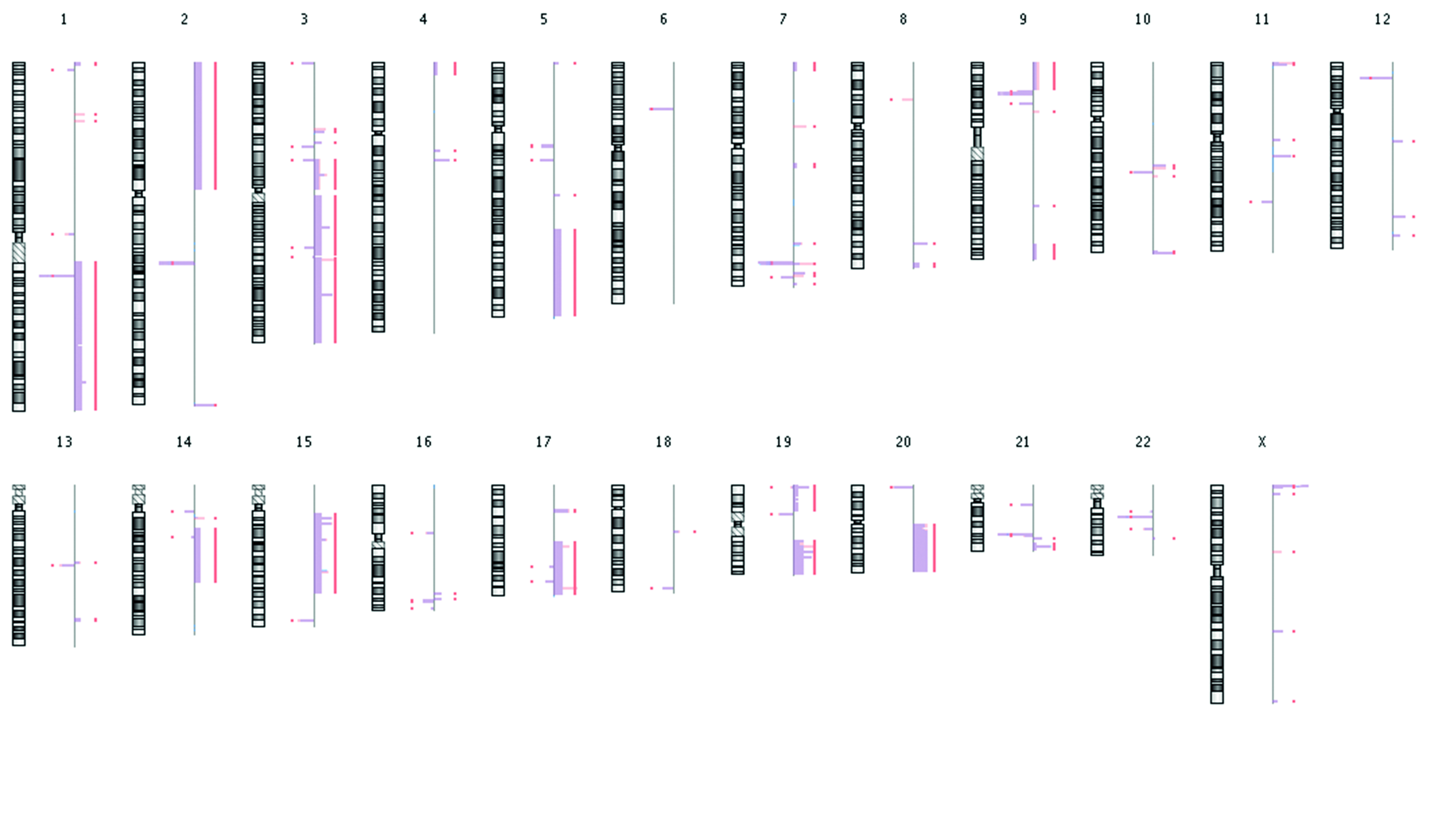

Supplement: Figure S1 — CGH analysis of CCH cells. (TIF) [file pone.0105099.s001.tif]

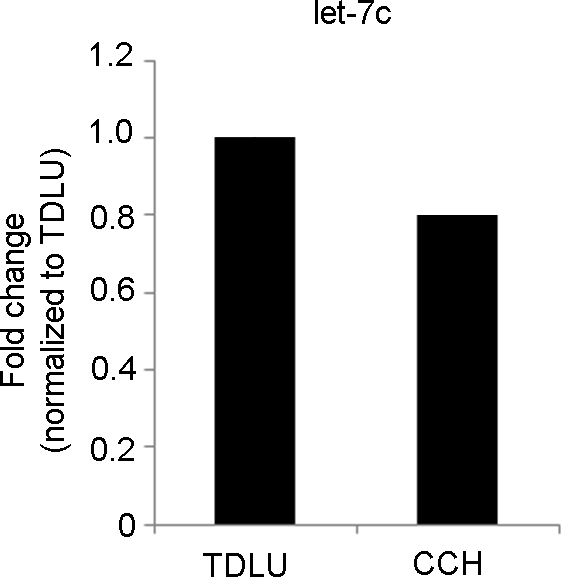

Supplement: Figure S2 — Validation of miRNA microarray results. The expression of let-7c was analysed in microdissected epithelial tissue from one additional patient using qRT-PCR. (TIF) [file pone.0105099.s002.tif]
